# Editorial: Chromatin Spatial Configuration and Function in Metazoans

**DOI:** 10.3389/fgene.2021.734981

**Published:** 2021-08-11

**Authors:** Paul Delgado-Olguín, Katarzyna Oktaba, Mayra Furlan-Magaril

**Affiliations:** ^1^Translational Medicine, The Hospital for Sick Children, Toronto, ON, Canada; ^2^Department of Molecular Genetics, University of Toronto, Toronto, ON, Canada; ^3^Heart & Stroke Richard Lewar Centre of Excellence, Toronto, ON, Canada; ^4^Unidad Irapuato, Centro de Investigación y de Estudios Avanzados del IPN, Irapuato, Mexico; ^5^Departamento de Genética Molecular, Instituto de Fisiología Celular, Universidad Nacional Autónoma de Mexico, Mexico, Mexico

**Keywords:** chromatin 3D architecture and dynamics, gene expression regulation, topologically associated domain (TAD), histone modifications, chromatin dynamics, non-coding RNA, repetitive (repeated) DNA, nuclear architecture

Discovering that heredity is encoded in DNA sparked relentless efforts to reveal the processes perpetuating the genetic code and deciphering how it is read and executed. Such efforts have shed light on the intricate interactions of multiprotein complexes with diverse regulatory elements in the DNA that dictate where and when genes are activated or shut down. DNA methylation, histone modification, chromatin accessibility, repetitive elements, non-coding RNAs, chromatin looping mediating interaction of distal regulatory elements, and structuring of chromatin into higher-order structures, add complexity layers to the molecular mechanisms behind transcriptional gene regulation. Genome-wide approaches applied to an ever-growing diversity of organisms, tissues, and cell types at multiple developmental stages and under different conditions are helping to progressively integrate these elements in a multilayered model of genome control. This integrative and evolving concept is now at the core of our understanding of the fundamentals of cellular identity and function, embryogenesis, homeostasis, and disease.

The present Research Topic includes 14 reports on key processes and regulators of chromatin modification and three-dimensional organization of the genome in the nucleus, and their link to gene control.

A broad perspective of genome organization is provided by Penagos-Puig and Furlan-Magaril, who walk us through discovery of the processes that control chromatin folding into heterochromatin and how its repressive activities are key for transcriptional dynamics. The authors then focus on recent findings suggesting that tethering heterochromatin domains toward the nuclear lamina instructs large-scale genome organization. Finally, they discuss evidence that altering such tethering is associated with aging disorders like progeria. This highlights functions of heterochromatin in multiple cellular processes (Allshire and Madhani, 2018). In this regard, Gerlitz summarizes emerging evidence of heterochromatin affecting the stiffness of the nucleus in response to mechanical forces like when the cell migrates through small pores, and in the regulation of gene expression programs promoting cell migration. Magaña-Acosta and Valadez-Graham, provide a more focused perspective of genome organization. They describe our current understanding of the mechanisms by which chromatin remodelers control the different levels of chromatin compaction and looping that dictate nuclear architecture. The authors also provide a close-up of how protein complexes including chromatin remodelers act on nucleosomes on specific promoters to promote transcription.

Given the close link between genome architecture and chromatin function with physiological processes (Mishra and Hawkins, 2017; Vilarrasa-Blasi et al., 2021), it is expected that elements of genome organization, e.g., topologically associated domains (TADs), are conserved in a wide range of metazoans (Harmston et al., 2017). This is exemplified by Lezcano et al., who summarize evidence of the three-dimensional genomic organization in mosquitoes. The authors highlight genomic features that vary in mosquito vs. other insects, suggesting that the evolution of such features might be constrained amongst related species. The authors also discuss the contribution of specific histone modifiers to chromatin looping and chromatin architecture to gene regulation.

Transmission of histone marks is an important component of epigenetic inheritance (Skvortsova et al., 2018; Tabuchi et al., 2018). Torres-Flores and Hernández-Hernández provide an overview of protamines, histones, histone modifications, and histone variants in the spermatid as potential markers of chromatin structure features that are retained in the mature sperm. The authors stress that understanding histone and histone modification retention in spermatids could have deep implications in inter and transgenerational inheritance. This would involve “readers” of histone marks like the chromodomain proteins (Cutter Dipiazza et al., 2021), as emphasized by DasGupta et al. The authors provide a comprehensive overview of the central function of chromodomain proteins as “readers” and nucleators of diverse protein and RNA complexes regulating gene expression and genome architecture locally and globally in *C. elegans*.

Specific combinations of histone marks are associated with transcriptional output (Bannister and Kouzarides, 2011), but their contribution is still not fully understood. Ntorla and Burgoyne describe the discovery of histone lysine crotonylation and the functions of the enzymes catalyzing and reading it. The authors also highlight its relevance in gene regulation for stem cell maintenance, and as a marker of metabolic status. Chromatin modification is prominently emerging as a key metabolic sensor (Suganuma and Workman, 2018). Asif et al. thoroughly break down the evidence linking metabolism with dynamic DNA and histone methylation, and histone acetylation. The evidence discussed points to changes to such epigenetic modifications as targets of diet on organ-specific and systemic metabolism.

A deeper understanding of the regulatory landscape genome-wide challenges the notion of “junk DNA.” Knowledge gaps of the function of some parts of the genome have been filled up by non-coding RNAs including long non-coding RNAs (lncRNAs) (Lee et al., 2019; Statello et al., 2021) and regulatory RNAs (Slack, 2006), which have functions in organizing genome architecture (Pisignano et al., 2019). Ramírez-Colmenero et al. highlight different mechanisms of action of lncRNAs with conserved sequence, genomic location, and structure, as regulators of gene expression by mediating chromatin looping, and formation of chromatin domains. Pérez-Molina et al. took a closer look at the regulation of lncRNAs. Their original research describes an Alu transposable element within the long non-coding RNA *Linc00441* that attenuates the expression of its host gene. Morf et al. discuss a different mode of action of regulatory RNAs, in which burst of the expression of regulatory RNAs containing protein binding motifs could favor concentration of protein factors for regulating nuclear processes locally.

Repeat elements were also considered “junk DNA,” but they are important regulators of the spatial configuration of the genome (Slotkin and Martienssen, 2007). This is exemplified by original research by Konkova et al. They found that the length of the repeat in the *1Q12* locus determines its repositioning relative to the nucleolus toward the center of the nuclei in response to ionizing radiation. Interestingly, the authors suggest that susceptibility to oxidative stress in response to radiation favors cells with shorter repeats and higher expression of satellite DNA in lymphocytes of patients affected by schizophrenia.

The function of chromatin modifiers and genome architecture in developmental processes or in physiology are the subject of active research (Van Der Weide and De Wit, 2019; Tan et al., 2021). Hernández-Hernández et al. discuss a multilayered regulatory network in which myogenic transcription factors interact with histone modifiers and genome organizers like CTCF to modify chromatin accessibility and mediate the interaction of distal elements, e.g., promoter-enhancer to turn on skeletal muscle gene expression. Yuan et al. provide an overview of cardiogenic transcription factors and how their activity is coordinated via interaction with enhancers to establish the logic of cardiac cell differentiation. The authors then engage in lively discussion of the evolutionary origin of enhancers and the use of genome-wide chromatin features coupled to multi-species alignment as tools for their identification. This collection of expert contributions highlights the outstanding progress being made toward our understanding of genome regulation. Constant technological and conceptual leaps in single-cell genomics, proteomics, and mutagenesis predict a more integrative and in-depth insight into the fundamentals of life.

## Author Contributions

MF-M, the guest editor, and the co-editors KO and PD invited expert contributions and handled peer review. PD wrote the Editorial with input from MF-M and KO. All authors contributed to the article and approved the submitted version.

## Conflict of Interest

The authors declare that the research was conducted in the absence of any commercial or financial relationships that could be construed as a potential conflict of interest.

## Publisher's Note

All claims expressed in this article are solely those of the authors and do not necessarily represent those of their affiliated organizations, or those of the publisher, the editors and the reviewers. Any product that may be evaluated in this article, or claim that may be made by its manufacturer, is not guaranteed or endorsed by the publisher.
